# Evaluation of CTLA-4 and PD-L1 Expression in Thyroid Carcinoma and Its Prognostic Significance

**DOI:** 10.7759/cureus.67004

**Published:** 2024-08-16

**Authors:** Nidhi Anand, Pallavi Srivastava, Nuzhat Husain, Deeksha Agarwal, Anurag Gupta, Roma Pradhan

**Affiliations:** 1 Pathology, Dr. Ram Manohar Lohia Institute of Medical Sciences, Lucknow, IND; 2 Endocrine Surgery, Dr. Ram Manohar Lohia Institute of Medical Sciences, Lucknow, IND

**Keywords:** anaplastic thyroid carcinomas, follicular carcinoma of the thyroid, immunotherapy, immunohistochemistry(ihc), immune proportion scores, tumour proportion scores (tps), ptc, cytotoxic t-lymphocyte-associated antigen 4, programmed death-ligand 1, immune-checkpoint inhibitors

## Abstract

Introduction

Immune checkpoint inhibitors (ICIs) targeting cytotoxic T-lymphocyte-associated antigen-4 (CTLA-4) and programmed death-ligand 1 (PD-L1) have revolutionised treatment and improved outcomes in various malignancies. We aimed to evaluate CTLA-4 and PD-L1 immunoexpression in thyroid tumours and correlated them with clinicopathological parameters.

Methods

The study included 90 cases of thyroid malignancies comprising papillary thyroid carcinoma (PTC) (n = 64, 54.2%), follicular thyroid carcinoma (FTC) (n = 19, 16.1%), anaplastic thyroid carcinoma (ATC) (n = 3, 2.5%), and poorly differentiated carcinoma (n = 4, 3.4%), two cases (1.69%) of non-invasive follicular thyroid neoplasm with papillary-like nuclear features (NIFTP) along with 26 cases (22%) of benign thyroid lesions. CTLA-4 (UMAB249) and PD-L1 (SP263) expression were assessed in all the cases of thyroid tumours. Results were compared with clinicopathologic parameters and overall survival.

Results

PD-L1 was positive in all three cases of anaplastic thyroid carcinoma (ATC), 33% (n = 21) cases of PTC, and 16% (n = 3) cases of FTC. PD-L1 positivity was significantly associated at tumour proportion score (TPS) ≥1% with lymphovascular invasion and age ≤40 years and at TPS ≥50% with tumour necrosis and N-stage. Immune proportion score (IPS) did not correlate with any clinicopathological parameters except for the N-stage. CTLA-4 was positive in six cases of PTC (1-5%); five showed lymph node involvement (p = 0.032). IPS was positive in 14 cases, and a significant association was seen with lymph node metastasis, lymphocytic infiltration, and lymphovascular invasion. Three cases of PTC showed co-expression for PD-L1 and CTLA-4 in tumour cells. No significant association was seen between PD-L1 expression and survival.

Conclusion

The current data suggest that PD-L1 is expressed in differentiated thyroid carcinoma, mainly PTC and ATC, indicating higher responsiveness to immunotherapy. A subset of PTC showed co-expression of PD-L1 and CTLA-4. These findings suggest the need for further investigation to utilise combinational immunotherapy, including anti-PD-L1 and anti-CTLA-4.

## Introduction

The tumour’s capacity to avoid immune destruction by the production of transformed cells that may resist immune surveillance by expressing inhibitory or stimulatory immune checkpoints on the surface of tumour cells and tumour-infiltrating lymphocytes (TILs) has been explored largely in the last few years. This led to the development of several FDA-approved immune checkpoint inhibitors (ICIs) for various malignancies, including melanoma [1], non-small cell lung carcinoma (NSCLC) [2], head and neck squamous cell carcinoma [3], and microsatellite unstable colorectal carcinoma [4]. Regarding the thyroid lesions, the programmed death-1/programmed death-ligand 1 (PD-1/PD-L1) axis has been explored in benign and malignant thyroid lesions, including differentiated, poorly differentiated and anaplastic malignancies. They have shown variable immunoreactivity to the checkpoint molecules; however, their diagnostic role is yet to be established [5].

PD-L1 is a B7 gene family member that is expressed on the tumour cells with its ligand PD-1 and acts synchronously to evade immune surveillance. Among differentiated thyroid carcinoma (DTC), papillary thyroid carcinoma (PTC) has shown increased expression of PD-L1 [6].

The management of PTC usually includes surgery and radioiodine therapy with a good response rate; however, a subset of PTC (10-30%) shows recurrence and metastasis on long-term follow-up. Hence, exploring the PD-1/PD-L1 axis in such cases might be helpful for second-line therapy. In addition to this, ATC, which is an aggressive cancer and is unresponsive to radioiodine therapy, has shown significant expression of PD-L1 immunohistochemistry (IHC) [7]. Hence, exploring ICIs among aggressive thyroid tumours may carve a path for better patient management and reducing mortality.

In the era of personalised medicine, studies depicting the expression of more than one immune checkpoint molecule are required, which may provide relevant information for the implementation of combinational ICI therapies for tumours with poor therapy response and aggressive tumours. Hence, we studied another ICI, T-lymphocyte-associated antigen-4 (CTLA-4). This is an immunoregulatory molecule that suppresses the proliferation and activation of T-lymphocytes. The antigen-presenting cells (APCs) expressing CD80 and CD86 on their surface interact competitively with CD28 or CTLA-4 receptors and stimulate co-stimulatory or inhibitory signals to T-cells. In terms of health, CTLA-4 helps maintain homeostasis and self-tolerance of the immune system. In malignancies, CTLA-4 expressed by tumour cells is supposed to facilitate tumour cell evasion by ablating the immune surveillance of immune cells (ICs) around the tumour because CTLA-4 inhibition results in increased activation of the immune system. Ipilimumab, an inhibitor of CTLA-4, was approved for the treatment of advanced or unresectable melanoma [8]. Our study is one of the first to evaluate the protein expression of CTLA-4 by IHC in benign and malignant thyroid lesions, along with the evaluation of PD-L1.

CTLA-4 and PD1/PD-L1 exert inhibitory effects on T-cell activity by differing mechanisms. CTLA-4 acts in the initial stages, while the PD-1/PD-L1 pathway acts on the activated T-cells. The two immunomodulators have been shown to interact, and anti-PD-L1 therapy reduces the tumour cell glycolytic activity. Tumours with low glucometabolic levels may benefit from anti-CTLA-4 therapy; hence, a combination of anti-CTLA-4 and anti-PD1/PD-L1 therapies may have a synergistic effect in the treatment of advanced malignancies [9].

We evaluated the tumour proportion scores (TPSs) and immune proportion scores (IPSs) for CTLA-4 and PD-L1 immunoexpression in thyroid tumours and correlated them with clinicopathological parameters. A comparative analysis of the co-expression of both the immunoregulatory molecules was also performed, with a detailed analysis of cases showing the co-expression of both markers and the survival analysis.

## Materials and methods

This retrospective and prospective case series was conducted in the Department of Pathology at a tertiary care hospital over 24 months from December 2019 to December 2021. For the retrospective part of the study, cases from January 2014 to November 2019 were included, and for the prospective part of the study, cases from December 2019 to August 2021 were included. In all, 118 cases were included based on the sample size calculation formula proposed by Daniel (1999) [10]. The Institutional Research and Institutional Ethical Committee (IEC 27/19) clearance was obtained from Dr. Ram Manohar Lohia Institute of Medical Sciences, Lucknow, India. Informed consent was obtained from all the prospective cases, and a waiver of consent was obtained from all retrospective cases.

Of 118 cases included in the study, 90 cases of malignant thyroid nodules (e.g., papillary, follicular, and anaplastic carcinomas), two cases of non-invasive follicular thyroid neoplasm with papillary-like nuclear features (NIFTP), and 26 cases of benign thyroid lesions (control group) were included. The histological specimens were in the form of total thyroidectomy (n = 66), hemithyroidectomy (n = 42), lobectomy (n = 7), and biopsy from the thyroid lesion (n = 3) with or without lymph node resections. The resection specimens were categorised according to the current WHO classification of thyroid malignancy [11]. A basic panel of IHC, including thyroglobulin, thyroid transcription factor-1 (TTF-1), pan-cytokeratin, and calcitonin, was used in poorly differentiated or anaplastic carcinomas.

A detailed clinical history, including age, sex, complete demographic profile, and radiological details, was documented. The overall survival (OS) was calculated for all the cases. The OS was defined as the time window between diagnosis and death or the last follow-up.

Inclusion and exclusion criteria

All the specimens of thyroid resections/nodules, where the diagnosis of neoplastic thyroid was rendered, were included. Largely necrotic and inadequate biopsies, autolysed specimens, and cases with insufficient clinical details were excluded from the study.

Histopathological evaluation

Haematoxylin and eosin (H&E)-stained slides from all the cases were reviewed by two pathologists (NA and NH) to confirm the histological diagnosis. Tumour site, size, the status of the capsule, pattern of growth, level of invasion, nodal metastasis, TNM-stage, and histomorphological parameters, including nuclear features, lymphovascular invasion, perineural invasion, presence of ICs, mitosis, and necrosis, were noted.

Immunohistochemical analysis

Immunohistochemical Expression, Assessment, and Scoring of PD-L1

PD-L1 IHC was performed using clone SP263 (Cat no. 7494190001) on a fully automated Ventana BenchMark XT (Ventana Medical System Inc., Tucson, AZ, USA). A positive control (human placenta) and negative control (obtained by omitting the primary antibody) were run with each batch.

All the PD-L1 stained sections were independently analysed for TPS and IPS by two authors (NA and NH). The TPS was assessed as a percentage of positive expression of PD-L1 on tumour cells, and the IPS was assessed as a percentage of PD-L1 expression on ICs. Cells with membranous PD-L1 expression were considered positive. The percentage of cells stained and the staining intensity were assessed. PD-L1 expression in terms of TPS and IPS was assessed at a cut-off of ≥1%, ≥10%, ≥25%, and ≥50%.

Immunohistochemical Expression, Assessment, and Scoring of CTLA-4

CTLA-4 IHC was performed using clone UMAB249 (Cat no. API11AA) (Diagnostic Biosystems, Pleasanton, CA, USA) at a dilution of 1:500. Tonsillar tissue was used as a positive control. The expression of CTLA-4 was assessed in both tumour cells and ICs. TPS and IPS scoring was done. The results were recorded based on the intensity of the staining reaction in the cytoplasm, as described below, as well as the estimated percentage of positive tumour cells [12].

Intensity 0: If there was no reaction in the cytoplasm. 

Intensity 1+: If there was a low number of cytoplasmic granules.

Intensity 2+: If there was a moderate number of cytoplasmic granules.

Intensity 3+: If there was a high number of cytoplasmic granules.

Statistical analysis

The statistical analysis was done using IBM SPSS Statistics, version 21.0 (IBM Corp., Armonk, NY, USA). The frequency of expression of PD-L1 and CTLA-4 in neoplastic thyroid lesions was assessed. Contingency tables and chi-square tests were used to correlate PD-L1 and CTLA-4 IHC results with tumour type, age, sex, pT stage, pN stage, ICs, capsular and vascular invasion, necrosis, and multiplicity. OS was compared using Kaplan-Meier estimates, and statistical significance was determined using the log-rank test. A multivariate analysis was also done. A value of p < 0.05 was statistically significant.

## Results

The study group included 118 cases of neoplastic thyroid lesions; 62% (n = 73) of the patients were aged ≤40 years. Most malignant cases were of PTC (n = 64, 54.2%) followed by follicular thyroid carcinoma (FTC) (n = 19, 16.1%). There were four poorly differentiated cases and three cases of ATC.

Correlation of clinicopathological parameter with PD-L1 expression in tumour

**Figure 1:**
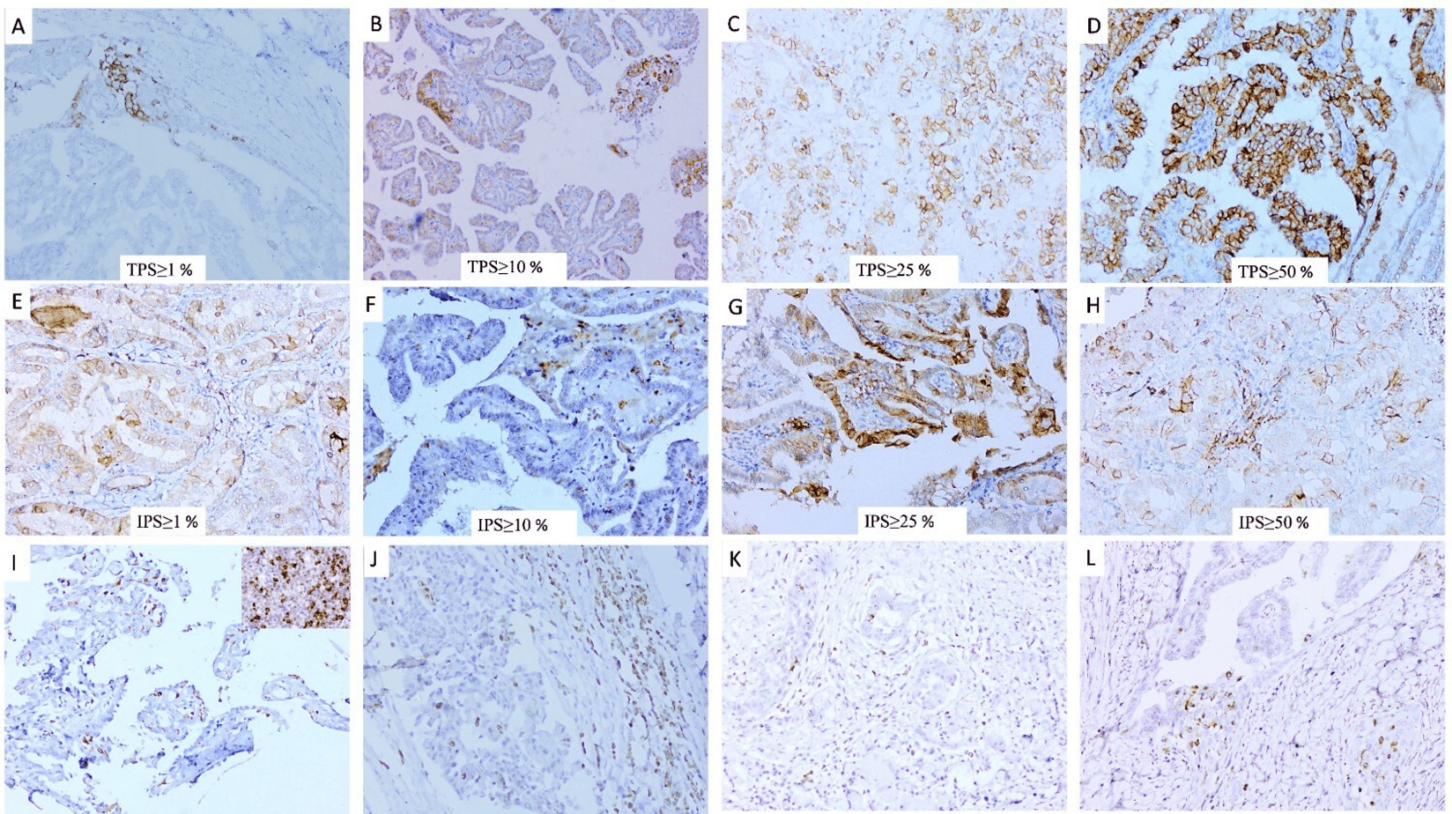
Immunohistochemical expression of PD-L1 and CTLA-4 in cases of thyroid malignancy. (A-D) PD-L1 expression in tumour cells at various cut-offs. (E-H) PD-L1 expression in tumour-infiltrating ICs at multiple cut-offs. (I) CTLA-4 expression in tumour cells is 2% (+++), and ICs are negative, with the inset showing positive expression in control (i.e., tonsil). (J) CTLA-4 expression in tumour cells is 5% (+++) and 30% (+++) in ICs. (K) CTLA-4 expression in tumour cells is 2% (+++) and 10% (++) in ICs. (L) CTLA-4 expression in tumour cells is 5% (+++) and negative in ICs. (A,I) DAB × 200; (B-H,J-L) DAB × 400

The association of TPS with demographic and clinicopathologic characteristics of the neoplastic thyroid nodules is shown in Table 1. An increase in the overall TPS was observed in malignant thyroid lesions compared to benign lesions, in size ≥2 than ≤2cm, in the unifocal lesions, and where LVI was evident. TPS was higher in tumours where necrosis and capsular breach were evident. TPS was 10.2% at a cut-off of ≥1%, 3.4% at a cut-off of ≥10 %, 2.5% at a cut-off of ≥25%, and 9.3% at a cut-off of ≥ 50%, as shown in Figures 1A-1D. PD-L1 positivity was significantly associated with lymphovascular invasion (p = 0.019) and age ≤40 years (p = 0.032) at TPS ≥1%, with tumour necrosis (p = 0.005) and N-stage (p = 0.012) at TPS ≥50%; however, no significant association was seen at the cut-offs of ≥10% and ≥25%. All three cases of ATC expressed PD-L1; however, none of the cases of poorly DTC expressed PD-L1.

**Table 1:** Correlation of clinicopathological parameters and TPS of PD-L1 in thyroid lesions TPS, tumour proportion score; DTC, differentiated thyroid carcinoma; T, tumour; LVI, lymphovascular invasion The chi-square test/Fisher’s exact test was applied as appropriate. *p-value <0.05 is considered significant.

Clinicopathological variables	No. of patients (%)	PD-L1 TPS ≥1%		PD-L1 TPS ≥10%		PD-L1 TPS ≥25%		PD-L1 TPS ≥50%	
		Positive (n = 12; 10.2%), no. (%)	p-value	Positive (n = 4; 3.4%), no. (%)	p-value	Positive (n = 3; 2.5%), no. (%)	p-value	Positive (n = 11; 9.3%), no. (%)	p-value
Age (years)									
≤40	73 (62)	4 (33.3)	0.032*	2 (50)	0.635	2 (66.7)	1.000	7 (63.6)	0.899
>40	45 (38)	8 (66.7)		2 (50)		1 (33.3)		4 (36.4)	
Gender									
Male	32 (27)	5 (41.7)	0.232	1 (25)	0.923	1 (33.3)	1.000	1 (9.1)	0.158
Female	86 (72.9)	7 (58.3)		3 (75)		2 (66.7)		10 (90.9)	
Capsular breach									
Evident	44 (37.2)	5 (41.7)	0.741	1 (25)	1.000	1 (33.3)	1.000	5 (45.5)	0.556
Not evident	78 (66.1)	7 (58.3)		3 (75)		2 (66.7)		6 (54.5)	
Diagnosis									
Benign	28 (23.7)	1 (8.3)	0.410	0	0.418	1 (33.3)	0.100	1 (9.1)	0.466
DTC	83 (70.3)	10 (83.3)		4 (100)		1 (33.3)		9 (81.8)	
Aggressive T.	7 (5.9)	1 (8.3)		0		1 (33.3)		1 (9.1)	
Tumour size (cm)									
≤2	27 (22.9)	3 (25)	0.983	2 (50)	0.418	0	0.578	2 (18.2)	0.728
>2 to 4	51 (43.2)	5 (41.7)		1 (25)		2 (66.7)		6 (54.5)	
>4	40 (33.9)	4 (33.3)		1 (25)		1 (33.3)		3 (27.3)	
T-stage									
T1 + T2	51 (43.2)	4 (40)	0.583	3 (75)	0.347	3 (100)	0.107	5 (55.6)	0.731
T3 + T4	39 (33.1)	6 (60)		1 (25)		0		4 (44.4)	
N-stage									
N0	12 (28.6)	4 (66.7)	0.227	2 (66.7)	0.412	2 (66.7)	1.000	1 (25)	0.012*
N1	30 (71.4)	2 (33.3)		1 (33.3)		0		3 (75)	
Focality									
Unifocal	99 (83.9)	11 (91.7)	0.440	4 (100)	1.000	3 (100)	1.000	9 (81.8)	0.844
Multifocal	19 (16.1)	1 (8.3)		0		0		2 (18.2)	
Tumor necrosis									
Present	19 (16.1)	1 (8.3)	0.440	1 (25)	0.509	1 (33.3)	0.412	5 (45.5)	0.005*
Absent	99 (83.9)	11 (91.7)		3 (75)		2 (66.7)		6 (54.5)	
TILs									
Present	54 (45.8)	7 (58.3)	0.356	3 (75)	0.331	2 (66.7)	0.592	5 (45.5)	0.983
Absent	64 (54.2)	5 (41.7)		1 (25)		1 (33.3)		6 (54.5)	
LVI									
Evident	51 (43.2)	9 (75)	0.019*	2 (50)	1.000	1 (33.3)	1.000	6 (54.5)	0.426
Not evident	67 (56.8)	3 (25)		2 (50)		2 (66.7)		5 (45.5)	
Outcome									
Expired	17 (14.4)	3 (25)	0.270	1 (25)	0.468	1 (33.3)	0.376	1 (9.1)	0.598
Survived	101 (85.6)	9 (75)		3 (75)		2 (66.7)		10 (90.9)	

PD-L1 expression in immune cells

The correlation of clinicopathological parameters and the IPS of PD-L1 in thyroid lesions has been compiled in Table 2. IPS was 23.7% at a cut-off of ≥1%, 9.3% at a cut-off of ≥10%, 3.4% at a cut-off of ≥25%, and 0.8% at a cut-off of ≥50% (Figures 1E-1H). IPS did not correlate with any clinicopathological parameters except for N-stage and lymphovascular invasion. However, overall IPS was significantly associated with age ≤40 years (p = 0.098), diagnosis (p = 0.052), tumour necrosis (p = 0.011), LVI (p = 0.056), and N-stage (p = 0.005).

**Table 2:** Correlation of clinicopathological parameters and IPS of PD-L1 in thyroid lesions TPS, tumour proportion score; IPS, immune proportion score; DTC, differentiated thyroid carcinoma; LVI, lymphovascular invasion. The chi-square test/Fisher’s exact test was applied as appropriate. *p-value <0.05 is considered significant.

Clinicopathological variables	No. of patients (%)	PD-L1 TPS ≥1%		PD-L1 TPS ≥10%		PD-L1 TPS ≥25%		PD-L1 TPS ≥50%	
		Positive (n = 28; 23.7%), no. (%)	p-value	Positive (n = 11; 9.3%), no. (%)	p-value	Positive (n = 4; 3.4%), no. (%)	p-value	Positive (n = 1; 0.8%), no. (%)	p-value
Age (years)									
≤40	73 (62)	15 (53.6)	0.301	5 (45.5)	0.239	2 (50)	0.635	1 (100)	1.000
>40	45 (38)	13 (46.4)		6 (54.5)		2 (50)		0	
Gender									
Male	32 (27)	9 (32.1)	0.494	3 (27.3)	0.990	1 (25)	1.000	0	1.000
Female	86 (72.9)	19 (67.9)		8 (72.7)		3 (75)		1 (100)	
Capsular breach									
Evident	44 (37.2)	10 (35.7)	0.844	6 (54.5)	0.214	1 (25)	1.000	1 (100)	0.373
Not evident	78 (66.1)	18 (64.3)		5 (45.5)		3 (75)		0	
Diagnosis									
Benign	28 (23.7)	4 (14.3)	0.294	1 (9.1)	0.124	0	0.418	0	0.808
DTC	83 (70.3)	23 (82.1)		8 (72.7)		4 (100)		0 (100)	
Aggressive T.	7 (5.9)	1 (3.6)		2 (18.2)				0	
Tumour size (cm)									
≤2	27 (22.9)	7 (25)	0.887	2 (18.2)	0.728	1 (25)	0.725	0	0.516
>2 to 4	51 (43.2)	11 (39.3)		6 (54.5)		1 (25)		1 (100)	
>4	40 (33.9)	10 (35.7)		3 (27.3)		2 (50)		0	
T-stage									
T1 + T2	51 (43.2)	11(45.8)	0.790	6 (66.7)	0.236	2 (50)	1.000	1 (100)	0.481
T3 + T4	39 (33.1)	13 (54.2)		3 (33.3)		2 (50)		0	
N-stage									
N0	12 (28.6)	12 (75)	0.268	4 (80)	1.000	0	0.024*	0	0.160
N1	30 (71.4)	4 (25)		1 (20)		2 (100)		1 (100)	
Focality									
Unifocal	99 (83.9)	24 (85.7)	0.765	9 (81.8)	0.844	3 (75)	0.509	1 (100)	1.000
Multifocal	19 (16.1)	4 (14.3)		2 (18.2)		1 (25)		0	
Tumor necrosis									
Present	19 (16.1)	6 (21.4)	0.380	3 (27.3)	0.290	2 (50)	0.121	1 (100)	0.161
Absent	99 (83.9)	22 (78.6)		8 (72.7)		2 (50)		0	
TILs									
Present	54 (45.8)	15 (53.6)	0.342	5 (45.5)	0.983	3 (75)	0.331	1 (100)	0.458
Absent	64 (54.2)	13 (46.4)		6 (54.5)		1 (25)		0	
LVI									
Evident	51 (43.2)	16 (57.1)	0.089	5 (45.5)	0.875	2 (50)	1.000	1 (100)	0.432
Not evident	67 (56.8)	12 (42.9)		6 (54.5)		2 (50)		0	
Outcome									
Expired	17 (14.4)	4 (14.3)	0.983	3 (27.3)	0.202	0	0.468	0	1.000
Survived	101 (85.6)	24 (85.7)		8 (72.7)		4 (100)		1 (100)	

PD-L1 expression in benign lesions and NIFTP

Twenty-eight controls of adenomas, including NIFTP, were analysed for PD-L1 expression in unremarkable follicular epithelial cells and infiltrating ICs. One of two NIFTP cases showed moderate (2+) expression in 40% of epithelial cells. ICs were expressed in both cases (5% and 1+). One case of Hurthle cell adenoma expressed strong (3+) staining in epithelial cells with moderate (2+) staining in ICs. Only one case of 24 follicular adenoma cases showed moderate (2+) expression in 2% of epithelial cells and 5% of ICs.

Correlation of clinicopathological parameter with CTLA-4 expression in tumour and ICs

Table 3 shows the correlation between clinicopathological parameters and tumour and IPS of CTLA-4 in thyroid lesions. Of 90 cases, six PTC cases showed positive expression for CTLA-4 with an intensity ranging from ++ to +++ and a percentage varying from 1-5%, as shown in Figures 1I-1L. Among them, five cases showed lymph node involvement (p = 0.032).

**Table 3:** Correlation of clinicopathological parameter and tumour and IPS of CTLA-4 in thyroid lesions TPS, tumour proportion score; IPS, immune proportion score; DTC, differentiated thyroid carcinoma; LVI, lymphovascular invasion The chi-square test/Fisher’s exact test was applied as appropriate *p-value <0.05 is considered significant.

Clinicopathological parameters	TPS CTLA4		p-value	IPS CTLA4		p-value
	Positive, N (%)	Negative, N (%)		Positive, N (%)	Negative, N (%)	
Age (years)						
≤40	4 (57.1)	69 (62.2)	1.000	7 (50.0)	66 (63.5)	0.330
>40	3 (42.9)	42 (37.8)		7 (50.0)	38 (36.5)	
Gender						
Male	3 (42.9)	29 (26.1)	0.334	8 (57.1)	24 (23.1)	0.007*
Female	4 (57.1)	82 (73.9)		6 (42.9)	80 (76.9)	
Diagnosis						
Benign	1 (14.3)	27(24.3)	0.615	0	28 (26.9)	0.084
DTC	27 (24.3)	77 (69.4)		13 (92.9)	70 (67.3)	
Aggressive T.	0	7 (6.3)		1 (7.1)	6 (5.8)	
Tumour size (cm)						
<2	3 (42.9)	24 (21.6)	0.233	6 (42.9)	21 (20.2)	0.158
2-4	3 (42.9)	50 (45.0)		4 (28.6)	47 (45.2)	
>4	3 (42.9)	37 (33.3)		4 (28.6)	36 (34.6)	
Capsular breach						
Evident	1 (14.3)	43 (38.7)	0.194	5 (35.7)	39 (37.5)	0.897
Not evident	6 (85.7)	68 (61.3)		9 (64.3)	65 (62.5)	
T-stage						
T1 + T2	3 (50)	35 (47.9)	0.923	8 (61.5)	30 (45.5)	0.289
T3 + T4	3 (50)	38 (52.1)		5 (38.5)	36 (54.5)	
N-stage						
N0	2 (100)	61 (83.6)	1.000	4 (66.7)	59 (85.5)	0.255
N1	0	12 (16.4)		2 (33.3)	10 (14.5)	
Focality						
Unifocal	7 (100)	92 (82.9)	0.232	12 (85.7)	87 (83.7)	0.844
Multifocal	0	19 (17.1)		2 (14.3)	17 (16.3)	
TILs						
Present	5 (71.4)	49 (44.1)	0.160	10 (71.4)	44 (42.3)	0.040*
Absent	2 (28.6)	62 (55.9)		4 (28.6)	60 (57.7)	
LVI						
Present	5 (71.4)	46 (41.4)	0.120	10 (71.4)	41 (39.4)	0.023*
Absent	2 (28.6)	65 (58.6)		4 (28.6)	63 (60.6)	
Tumour necrosis						
Present	0	19 (17.1)	0.232	2 (14.3)	17 (16.3)	0.844
Absent	7 (100)	92 (82.9)		12 (85.7)	87 (83.7)	
Outcome						
Expired	0	5 (4.5)	0.566	1 (7.1)	4 (3.8)	0.565
Survived	7 (100)	106 (95.5)		13 (92.9)	100 (96.2)	

CTLA-4 expression was also evaluated in ICs, and 14 cases showed expression in ICs, which correlated with clinicopathological parameters. A significant association with lymph node metastasis (p = 0.025) was found, along with lymphocytic infiltration (p = 0.04) and LVI (p = 0.023). One case of follicular adenoma also showed CTLA-4 expression (10% ++).

Correlation of PD-L1 and CTLA-4 co-expression in tumour and co-expression of PD-L1 in tumour/CTLA-4 in TILs

**Figure 2:**
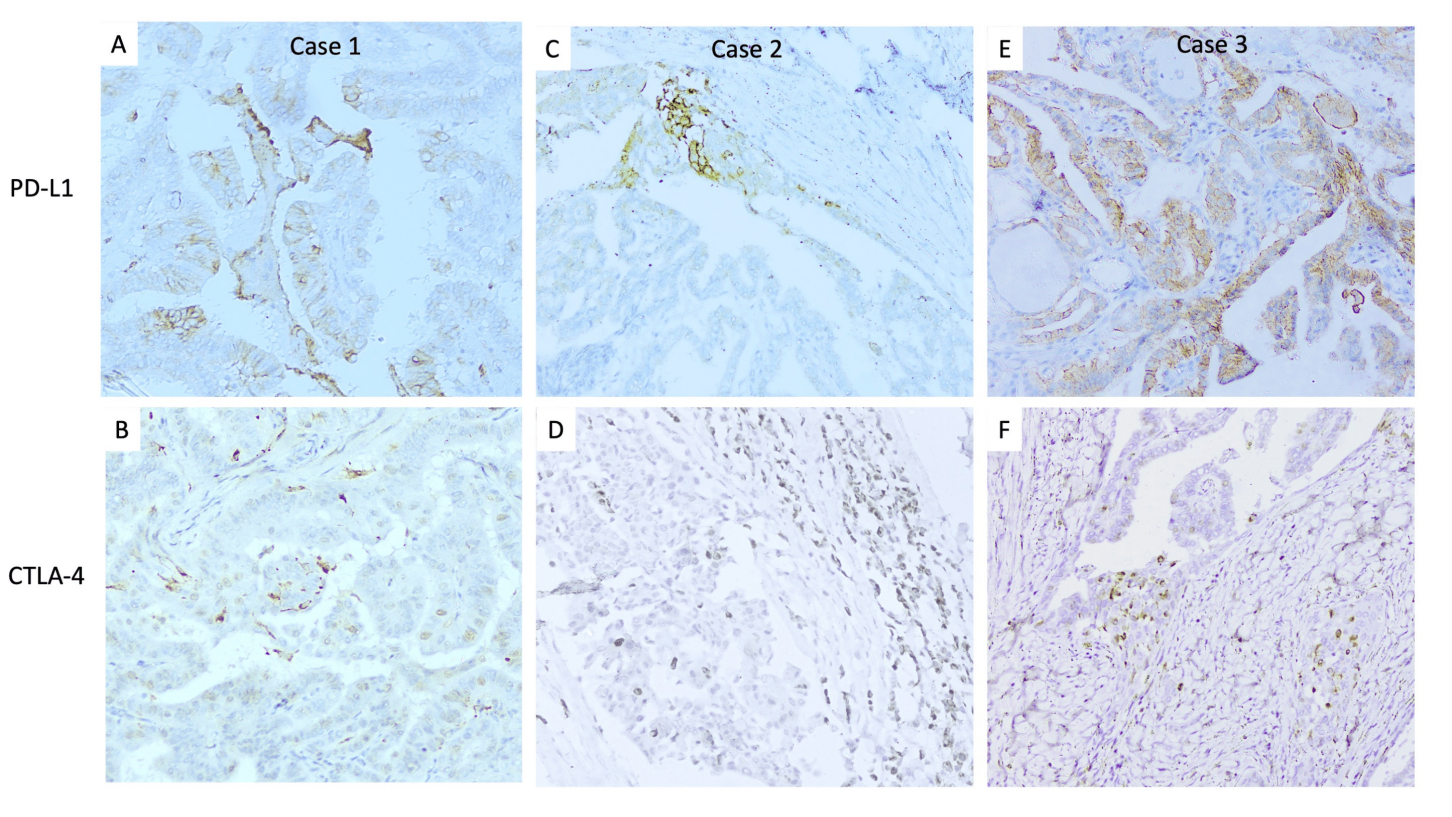
Papillary thyroid carcinoma cases showing combined expression for PD-L1 and CTLA-4 in tumour cells (A,B) Case 1 shows 20% (+++) of PD-L1 and 10% (+++) of CTLA-4 expression in tumour cells. (C,D) Case 2 shows 10% (+++) of PD-L1 and 2% (+++) of CTLA-4 expression in tumour cells. (E,F) Case 3 shows 80% (+++) of PD-L1 and 2% (+++) of CTLA-4 expression in tumour cells. (A-F) DAB × 400

Among seven cases showing positivity for CTLA-4, three showed co-expression of PD-L1, as depicted in Figure 2. Among 14 cases showing positivity for CTLA-4 in ICs, a total of six cases (five PTC and one ATC) showed co-expression for PD-L1 in tumour cells.

Univariate and Multivariate Survival Analysis

**Figure 3:**
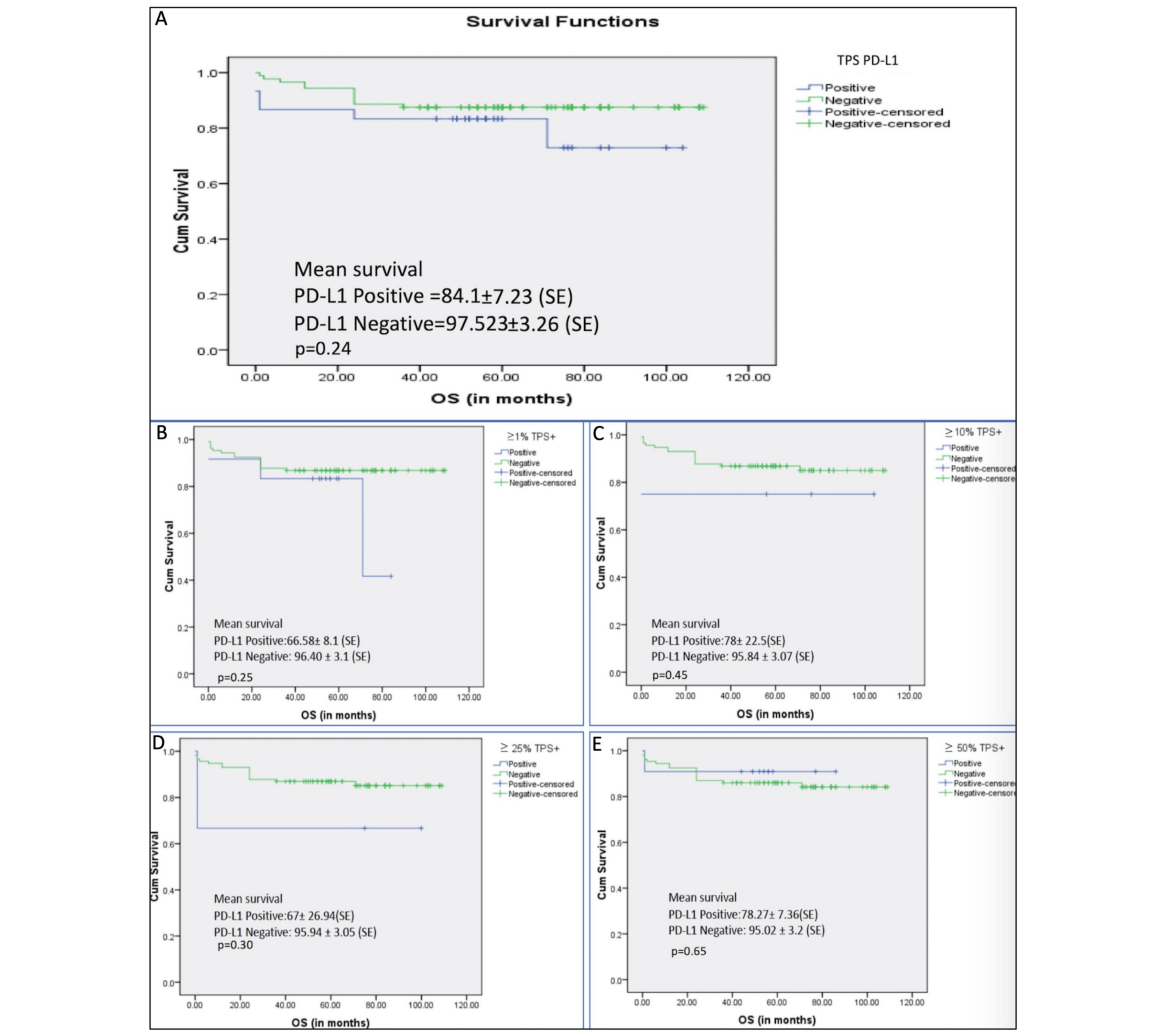
Kaplan-Meier estimates of overall survival (A) Overall survival of the cases with PD-L1 positive versus PD-L1 negative in tumour cells. (B) PD-L1 TPS ≥1%. (C) PD-L1 TPS ≥10%. (D) PD-L1 TPS ≥25%. (E) PD-L1 TPS ≥50%.

The mean of OS was 61.6 ± 26.24 (0-109 months) months. Using univariate analysis, the factors that influenced short-term survival included age (>40 yrs), histologic type (aggressive tumour type), capsular breach, and PD-L1 expression, as illustrated in Figure 3 and Table 4. Table 5 shows the multivariate analysis using the above as covariates; only histologic type (p < 0.05) was significant.

**Table 4:** Univariate survival analysis of PD-L1 expression and other covariates with overall survival as the outcome TPS, tumour proportion score; IPS, immune proportion score; FTC, follicular thyroid carcinoma; PTC, papillary thyroid carcinoma; LVI, lymphovascular invasion A log-rank (Mantel-Cox) test was applied. *p-value <0.05 is considered significant.

Clinicopathological variables	Number of patients	Mean survival (months ± SE) (95% CI)	p-value
Age (years)			
≤40	73	106.50 ± 1.74 (103, 110)	<0.05*
>40	45	73 ± 6.4 (60.38, 85.47)	
Histological types			
Differentiated thyroid carcinoma (PTC/FTC)	83	94.58 ± 3.70 (87.33, 101.84)	0.001*
Aggressive carcinoma	7	36.714 ± 13.49 (10.25, 63.17)	
Capsular breach			
Evident	44	81.39 ± 5.76 (70, 92.7)	0.039*
Not evident	74	100.39 ± 3.12 (94.27, 106.51)	
LVI			
Evident	51	91.47 ± 5.01 (81.64, 101.31)	0.384
Not evident	67	97.47 ± 3.86 (89.90, 105.04)	
T-stage			
T1 + T2	38	99.531 ± 4.60 (90.50, 108.56)	0.151
T3 + T4	41	86.58 ± 6.35 (74.13, 99.03)	
N-stage			
N0	63	95.20 ± 0.28 (86.8, 103.61)	0.56
N1	12	101 ± 6.70 (87.86, 114.13)	
PD-L1 TPS (≥1%)			
Positive	30	84.09 ± 7.23 (69.91, 98.27)	0.24
Negative	88	97.52 ± 3.26 (91.12, 103.91)	
PD-L1 IPS (≥1%)			
Positive	44	87.01 ± 5.46	0.32
Negative	74	97.81 ± 3.52	

**Table 5:** Multivariate Cox regression model for different variables CI, confidence interval; HR, hazard ratio; T, tumour; LVI, lymphovascular invasion HR and its 95% confidence interval calculated from the Cox regression model *p-value <0.05 is considered significant.

Covariates	p-value	HR	95.0% CI for HR	
			Lower	Upper
Differentiated carcinoma vs aggressive T.	0.012*	8.818	1.628	47.749
Age	0.734	6.18	0	5.02
T-stage	0.069	4.912	0.881	27.374
N-stage	0.292	0.291	0.029	2.897
Capsular invasion	0.613	1.593	0.263	9.645
Necrosis	0.824	1.189	0.258	5.477
LVI	0.419	1.925	0.939	9.426

## Discussion

Reducing tumour growth and achieving complete remission are great challenges, and the field of cancer immunotherapy has shown a ray of hope in combination with other treatment modalities. Cancer immunotherapy and ICIs are rapidly advancing and are considered potent therapeutic strategies for aggressive cancers that are unresponsive to treatment. Immunotherapy is also being considered for thyroid cancers. The role of PD-L1 in thyroid malignancies has been studied; however, the role of CTLA-4 needs to be explored [7-9]. Most of the studies on PD‑L1 expression in various follicular cell-derived thyroid carcinomas have used TPS and CPS with a cut-off of 1% and 5%; only Cantara et al. used a cut-off of 25% of TPS to assess PD-L1 immunoexpression in ATC [13].

Various clones of PD-L1 have been tested in thyroid carcinoma; the most commonly used clones were SP142 and SP263, followed by 22C3, E1L3N, and 28-8 [7,13-16]. We used clone SP263 for PD-L1 immunostaining in the current study. As there are no specific guidelines for the type of clone as well as for the cut-off points for TPS/CPS scoring for PD-L1 expression for thyroid carcinoma, unlike other carcinomas, such as lung, breast, and urothelial carcinoma, we evaluated PD-L1 at various cut-off values of ≥1%, ≥10%, ≥25%, and ≥50%. We also performed IHC on the whole tissue sections instead of tissue microarray (TMA) blocks that contain tissue cores of multiple cases in a single block. It helped to avoid intratumoral heterogeneity.

The diagnostic role of PD-L1 in thyroid carcinoma is not yet established; Cunha et al. studied 293 DTC cases and benign nodules and concluded that PD-L1 has no role in distinguishing benign from malignant cases [17]. More recently, Fu et al. studied NIFTP (52 cases), benign nodules (40 cases), and encapsulated variant of PTC and concluded that PD-L1 helped distinguish NIFTP/benign from an encapsulated variant of PTC [5]. In the current study, we found increased expression in DTC than NIFTP and benign nodules, while 100% ATC showed PD-L1 expression, consistent with the finding of Chowdhury et al., which demonstrated a significant positive expression of PD-L1 (clone E1L3N) in the aggressive variant [18]. The findings were not statistically significant; hence, a diagnostic role was not established. However, it may be concluded that PD-L1 might help differentiate between benign/NIFTP and malignant thyroid lesions. Chowdhury et al. also found a significant correlation between PD-L1 expression and shorter disease-free survival (DFS) [18]. Though, in the current study, DFS was not determined, we found that PD-L1 immunoexpression at a cut-off of ≥50% was significantly associated with the parameters, which are associated with a poor clinical outcome like lymph nodal metastasis and tumour necrosis.

PD-L1 has been proven to have a prognostic significance in various cancer types; however, its prognostic significance in thyroid carcinoma is yet to be established. A meta-analysis conducted by Girolami et al. [19] and Aghajani et al. [20] found that PD-L1 positivity in DTC was significantly associated with lower DFS.

We did not evaluate DFS; however, among malignant cases, 41% (37 of 90) cases showed disease recurrence, and among them, 30% (11 of 37) showed PD-L1 expression. On pooling the findings of four studies, Girolami et al. concluded that PD-L1 expression has no role in OS. Our study also found no significant association of PD-L1 expression with OS; however, we found that PD-L1-positive thyroid carcinoma cases had lower OS [19]. In our study, the survival analysis was also done at various cut-off values for PD-L1, though the findings were not statistically significant. The cases with ≥1% and ≥25% PD-L1 expression had lower OS. 

CTLA-4 immunoexpression in thyroid carcinoma has not been studied. There are only very few studies on cell lines and tumour cells using CTLA-4 receptor ligands, including CD80 and CD86 [21]. This is the first study on CTLA-4 expression by IHC in tumour and ICs in thyroid carcinoma. However, the role of CTLA-4 has been extensively studied in various other malignancies, including lung, breast, gastric, colorectal, and malignant melanoma [22-25]. Anti-CTLA-4 therapy is one of the first immunomodulators in the field of immunotherapy, but in recent times, the studies on CTLA-4 receptors have been limited due to the immune-related adverse events that occurred in patients who were on single-line therapy of anti-CTLA-4. With the advent of more effective and less harmful immunotherapy, including anti-PD-1 and anti-PD-L1 treatment, the use of anti-CTLA-4 has been reduced; however, combinational therapies, including anti-CTLA-4 with PD-1 or PD-L1, are being administered and shown promising results, as the elevated PD-L1 expression is often associated with poorer prognosis in several cancers, including thyroid carcinoma. It can indicate a more aggressive disease course and help predict response to certain therapies. However, its utility in thyroid malignancy is still not approved due to the unavailability of research directed toward it.

The prognostic role of CTLA-4 in thyroid carcinoma is still controversial and under evaluation, while in NSCLC, its expression was found to be related to decreased death rates. However, in breast carcinoma, contradictory results were seen, as a high level of CTLA-4 mRNA expression correlated with higher stage and lymph node metastasis [22,26]. In our study, among six CTLA-4 positive cases, five had lymph node metastasis, and one patient died of the disease; however, due to the limited number of positive cases in our study, the findings are not conclusive.

A meta-analysis by Hu et al. concluded a significant correlation between CTLA-4 and OS in nasopharyngeal carcinoma, malignant hematologic diseases, and glioblastoma, most of which had a strong pooled hazard ratio (HR). In contrast, malignant pleural mesothelioma showed a favourable effect of CTLA-4 overexpression [27]. In thyroid carcinoma, the correlation between OS and CTLA-4 needs to be explored, and studies with larger cohorts need to be conducted to understand the prognostic role of CTLA-4 [27].

In our study, an increased expression of PD-L1 was seen in DTC and anaplastic carcinoma; however, the prognostic role of PD-L1 was not found. CTLA-4 expression was seen in only 6.6% of cases, and three PTC cases showed co-expression for both markers and can be potential targets for combinational immunotherapy. The FDA has approved various combinational therapies for malignancies, including NSCLC, advanced renal cell carcinoma, or advanced melanoma [28]. PD-L1 and CTLA-4 inhibitor combination is currently being tested in thyroid malignancies with two ongoing clinical trials. The DUTHY trial is testing the efficacy of combinational therapy, i.e. anti-PD-L1 (durvalumab) + anti-CTLA-4 (tremelimumab), in advanced and refractory differentiated and ATC with promising results [29]. Another ongoing clinical trial in phase I is testing durvalumab (MEDI4736) with tremelimumab in combination with image-guided stereotactic body radiotherapy (SBRT) for treating metastatic ATC [30]. Hence, we recommend testing multiple ICIs to help clinicians better manage the unresponsive advanced cases of differentiated thyroid malignancies. 

We also studied CTLA-4 expression in TILs; 14 cases showed CTLA-4 expression, which correlated with positive LVI and nodal metastasis. These findings were supported by the study conducted by Guo et al., in which CTLA-4 in TILs was found to be associated with aggressive clinicopathologic features, including larger tumour size, lymph node metastasis, and high TNM in intrahepatic cholangiocarcinoma, and stated that overexpression of CTLA-4 in TILs promotes the invasion and metastasis [31].

Guo et al. also analysed the expression of PD-L1 in tumours and CTLA-4 in TILs and found that the co-expression had better sensitivity in predicting OS and recurrence [31]. We also analysed these cases, among which 6.5% cases (five PTC and one ATC) showed co-expression of PD-L1 (in tumour) and CTLA-4 (in TILs), though these cases showed clinicopathologic findings associated with disease progression, including capsular breach (four of six cases), tumour necrosis (five of six cases), lymph node involvement (four of six cases), and OS (three of six cases died of disease within six months of the diagnosis); however, due to the limited number of cases, the findings cannot be concluded. An increased expression of CTLA-4 in TILs has been observed, and its coexpression with PD-L1 in tumours might have a prognostic role in thyroid carcinoma; hence, more studies emphasising the correlation might be required to establish the prognostic role.

Limitations

The current study is a novel attempt to study the frequency of CTLA-4 and PD-L1 neoplastic thyroid lesions; however, there are certain limitations. The current study’s sample size was small, and the immunostaining results were not compared with any molecular technique, like ThyroSeq analysing a panel of genes for mutations and gene fusions, ordering detailed information about the tumour’s genetic landscape, and helping to predict prognosis and treatment responses. The study sample included only cases from a single tertiary care centre; therefore, the expression of CTLA-4 and PD-L1 IHC in neoplastic thyroid lesions across the different regions and ethnic groups could not be assessed. The number of cases of ATC and poorly DTC were very few to evaluate the utility of these markers in these lesions accurately.

## Conclusions

Future directions for CTLA-4-and PD-1/PD-L1-based immunotherapy in thyroid carcinoma, including combination therapy and individualised treatment, and understanding the complex mechanisms of combined therapy will continue to be explored. We propose testing for more than one checkpoint inhibitor testing, which might give new directions in thyroid immunotherapy and provide more effective, personalised, and targeted treatment options for patients.
